# Getting to the root of root–microbe interactions

**DOI:** 10.1177/00368504241278783

**Published:** 2024-09-05

**Authors:** William L King, Regan J Hayward, Marc Goebel, Suzanne M Fleishman, Taryn L Bauerle, Terrence H Bell

**Affiliations:** 1 School of Biological Sciences, 7423 University of Southampton, Southampton, UK; 2 Helmholtz Institute for RNA-Based Infection Research, 28336 Helmholtz Centre for Infection Research, Würzburg, Germany; 3 Department of Natural Resources and the Environment, 5922 Cornell University, Ithaca, NY, USA; 4 Department of Plant Science, 8082 The Pennsylvania State University, University Park, PA, USA; 5 School of Integrative Plant Science, 5922 Cornell University, Ithaca, NY, USA; 6 Department of Physical and Environmental Sciences, 33530 University of Toronto Scarborough, Toronto, ON, Canada

**Keywords:** Root morphology, root microbiome, root functionality, fine roots, spatial microbiome

## Abstract

Microbial relationships with roots influence many ecosystem functions and nutrient fluxes, including their sometimes-profound effects on plant health and productivity. Fine roots were often classified with a diameter less than 2 mm, but fine roots under that size perform distinct functional roles in the environment. Importantly, two broad functional categories of fine roots are *absorptive* and *transportive*, with absorptive fine roots acting as metabolic hotspots for root activity. In two of our recent studies, we have shown that several microbial community characteristics differ between absorptive and transportive fine roots, including composition, abundance, and function, as well as the root metabolome. This highlights a growing recognition within microbial ecology that we must consider fine-scale environmental variability, such as root physiology and morphology, when interpreting microbial patterns. In this commentary, we summarize the findings of our latest article, further speculate on some of these patterns, and suggest future studies for examining decomposition and applying cutting-edge single-cell sequencing techniques.

## Root functional roles and microbial relationships

Plants form intricate relationships with microorganisms.^1,2^ These relationships are encouraged, either directly or passively, by the release of carbon-rich compounds, called exudates, and other rhizodeposits into the surrounding soil environment.^3^ This zone of influence is often described as the “rhizosphere,” in which microbial composition, function, and abundance, are typically distinct from the surrounding bulk soil.^4^ The rhizosphere concept was first defined by Hiltner^5^ over 100 years ago and has been a substantial research area in recent decades, particularly with the improvements in next generation sequencing availability and financial accessibility. The rhizosphere is a gradient of plant influence, where fluctuations of a number of chemical, biological, and physical parameters are observed,^6^ generally less than 2.0 mm from the root surface. For example, oxygen, carbon dioxide, water, exudates, enzymes, and nitrogen and phosprous.^6^ In addition to the rhizosphere, roots also have distinct microorganisms that colonize the root surface (i.e. the rhizoplane) and the internal root environment (i.e. the endosphere).^7^ While we understand the impact of roots on microbial assembly in a general sense, we are still identifying the direct mechanisms of feedback between host and microbe, how those lines of communication may have evolved, and whether they can be manipulated. In particular, we understand little about impact of root morphology and functionality^8,9^ on root–microbial feedback, in part because of the relatively hidden nature of roots beneath the soil and in part because of the painstaking efforts that are required for fine-scale root separation.

For nearly a century, fine roots have been categorized into different roles based on root morphology and behavior.^10,11^ Despite this, one popular method of studying fine roots has been collecting all fine roots below an arbitrary diameter size (typically <2 mm) and considering them as functionally equivelant^8,12^ (eloquently illustrated in Figure 1 in Pregitzer (2002)). This “traditional diameter approach” has persisted in root ecology studies.^8,12,14^ While fine roots might be categorized in a variety of ways, based on traits such as shape, size, and age, they can be binned into two important and broad functional categories based on the role they play in the environment: absorptive fine roots and transportive fine roots.^8,15^ Absorptive fine roots are typically the key point of interaction between plants and the belowground environment and display greater metabolic flux and activity relative to transportive fine roots. In contrast, transportive fine roots show greater lignification and transport (i.e. xylem) capacity. The differences between absorptive and transportive fine roots cannot be understated. For example, when classifying fine roots according to functional classification instead of by diameter (<2 mm) net terrestrial primary productivity estimates were found to be overestimated by 30%.^8^ The separation of fine roots into functional categories can be performed by counting backwards from the most distally produced roots (see the morphometric approach in Freschet et al.^13^). As a general rule, roots that are root order 1 and 2 are typically the absorptive fine roots, while root orders 4 and above are the transportive fine roots.^8,9^ These categories are based on a suite of functional and morphological factors (see Figure 3 in McCormack et al.^8^).

## Paper summary and speculation

The potential importance of these root morphological and functional differences on microbiome recruitment inspired our recent publication, released in *Plant, Cell & Environment*.^1^ Most microbiologists and microbiome scientists studying root-microorganisms tend to collect roots (either all roots or those below a given size diameter) and homogenize them to create a composite sample.^16^ Understandably, it can be painstakingly laborious to separate out fine roots. Although, some efforts have been made to link root traits (e.g. diameter, length) to microbial composition,^17–19^ typically in relation to mycorrhizal fungi colonization,^14,18^ the fine roots are generally not sorted (i.e. a root cluster is homogenized) or DNA is collected from root tips and correlations applied between the microbiome and whole fine root clusters (i.e. <2 mm diameter).^14,19^

In this study, we separated the fine roots of four tree species based on assumed functional groupings (absorptive vs. transportive) and assessed the rhizoplane and rhizosphere microbiome, in parallel with the root metabolome. Overall, we detected an interaction between tree species and root functional type for both bacteria and fungi. At a finer scale, when we compared absorptive and transportive roots we observed: (i) rhizoplane, but not rhizosphere, bacterial compositional differences, (ii) differences in bacterial functions assigned to sugar and urea transport, and (iii) metabolome differences driven by sugars, amino acids and fatty acids. These findings reinforced our earlier findings that root functional type impacts microbial assembly.^20^ When we compared the rhizoplane and rhizosphere microbiome compartments for individual root functional groups, we observed differences for absorptive fine roots but not transportive fine roots.

**Figure 1.**
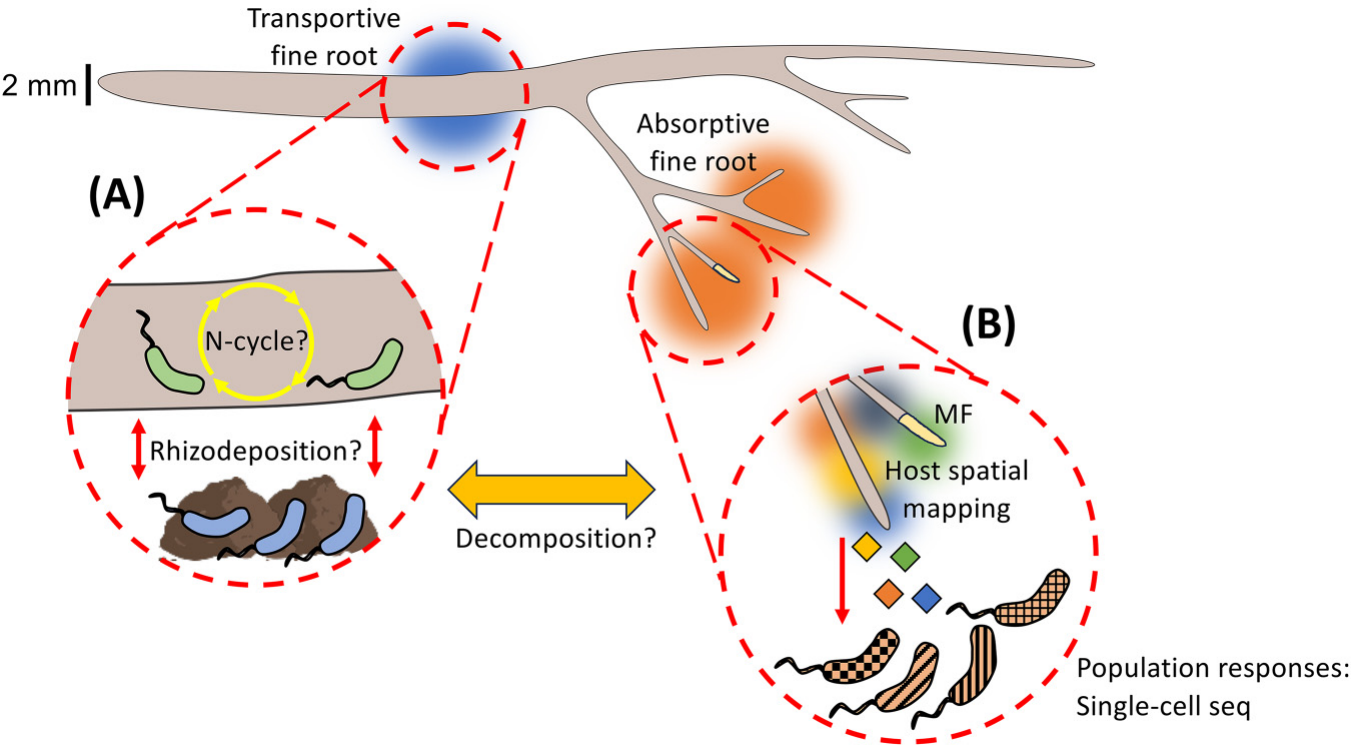
Conceptual figure of root–microbe interactions on (A) transportive and (B) absorptive fine roots and potential questions. MF is mycorrhizal fungi. Bacteria in (A) are root surface-associated bacteria (i.e. the rhizoplane). The blue and orange haze indicates the potential zone of influence each fine root exerts on the environment, and multiple colors in (B) indicate the diversity of exudate resources released by absorptive fine roots. A scale size is provided to indicate fine roots are typically below 2 mm in diameter, but for some tree species many fine roots may be less than 1 mm or 0.5 mm in diameter. For anatomical descriptions and illustrations for absorptive and transportive fine roots, see Freschet et al. (2021),^13^ McCormack et al. (2015),^8^ and Pregitzer (2002).^9,12^ Text within the figure links to speculation throughout the commentary article, including rhizodeposition from transportive fine roots, differences in N-cycling and decomposition, and applying cutting-edge sequencing techniques to spatially link root–microbial interactions.

Our most surprising finding was the lack of a significant difference in microbial composition between absorptive and transportive fine roots in the rhizosphere. We had expected that differences in metabolic flux from absorptive and transportive fine roots would lead to highly distinct rhizosphere microbial compositions. However, when we compared the rhizosphere and rhizoplane microbiome compartments for each root functional type, we found differences between the microbiome compartments for absorptive fine roots but not for transportive fine roots. Understandably, transportive fine roots are not exuding carbon, hence the similarity between the microbiome compartments, yet we did not detect a difference between the absorptive and transportive fine root rhizospheres. Given these patterns, we could speculate that the transportive fine root rhizosphere may represent a transitionary state that has been co-influenced by both root exudation and rhizosphere senescence, so we are unable to, compositionally, tease apart differences. We did not characterize microbial functions for the rhizosphere, so a lack of compositional difference does not necessarily indicate functional equivalency between those consortia. Our results highlight the need to include rhizosphere age and microbial activity, and events leading to the formation of rhizosphere in future experiments. Due to the lignification on transportive fine roots, the formation of the transportive fine root rhizosphere could be driven by rhizodeposition related to the formation of the outer bark and secondary growth,^8^ which may also explain similarities between the rhizosphere and rhizoplane (Figure 1). Future studies that leverage larger sample sizes and/or the inclusion of more tree species with varying root morphology may shed more light on the transportive fine root rhizosphere.

Although fine roots of different functional types did not differ in their rhizosphere microbial communities, the rhizoplane microbiome varied in composition and function between absorptive and transportive roots. It is difficult to decouple whether rhizoplane differences were primarily driven by root morphology and/or root exudation, but the overrepresentation of sugar transport systems in the absorptive fine root rhizoplane could suggest it is more related to exudation rather than root morphology. We observed an enrichment in urea transport and the amino acid ornithine for the transportive fine roots; urea and ornithine, in concert with arginine, can be important molecules for nitrogen cycling and storage.^21^ It is tempting to speculate that there may be distinct fine-scale nitrogen dynamics on transportive fine roots relative to absorptive fine roots, which may select for more copiotrophic bacteria. Nitrogen can also be important for decomposition processes. Thus, differences in microbial-facilitated nitrogen and carbon cycling according to fine root functionality may have further global implications for our understanding of C and N modelling in soils.

## Potential future directions—Decomposition and sequencing advances

The rates at which roots decompose have important implications for global C cycling. Given that absorptive fine roots are the metabolic hotspot of activity and lack secondary development,^8^ it would be natural to assume that they also decompose faster relative to transportive fine roots; however, transportive fine roots actually decompose faster.^22,23^ The lower decomposition of absorptive fine roots has been correlated to a higher acid insoluble fraction of C relative to transportive fine roots.^22^ Decomposition rate is driven by a combination of factors, including litter quality, edaphic factors, interactions with microorganisms, and climatic features.^24,25^ While we did not consider decomposition in our study, it could be possible that the existing microbiome could be important for early decomposition dynamics, before being taken over by soil saprotrophs. We may also speculate that the more similar a root microbiome is to the bulk soil microbiome, the greater the potential for soil saprotrophic diversity in the root microbiome and therefore effecting early decomposition rates. For example, we previously observed a greater microbiome similarity between the transportive fine root ectorhizosphere (labelled rhizoplane in that study) and bulk soil^20^ and in this study, a similarity between the transportive fine root rhizoplane and rhizosphere. Future studies could temporally track microbiome composition on absorptive and transportive fine roots and relate the microbial composition to decomposition rate, in particular to identify how existing colonizers, as opposed to microbial colonizers following root death, may influence decomposition. Identifying the microbial drivers of decomposition for disparate root types will be vital when modelling soil C dynamics. In terms of nitrogen cycling, N appears to accumulate in the transportive fine roots while it is rapidly lost in absorptive fine roots,^23^ and this N is suggested to not be available for plant uptake.^22^ Transportive fine roots also have higher C:N ratios (∼40–50)^8^ and decompose faster relative to absorptive fine roots.^22,23^ Given that we observed an enrichment of functions and metabolites related to N transport and storage for transportive fine roots, it would be prudent to identify how N is distributed during decomposition. Similarly, the higher C:N ratios of transportive fine roots may be stimulating nitrogen immobilization (or similar microbial feedback) which could explain the accumulation of nitrogen on transportive fine roots.

As we further investigate root–microbe interactions, progress can be made using technologies that can provide further spatial resolution and single-cell responses. Techniques such as spatial metatranscriptomics (SmT)^26^ and spatial host–microbiome sequencing (SHM-seq)^27^ currently allow for the quantification of host gene expression alongside microbial community profiling (i.e. 16S rRNA gene and ITS region). However, there are currently few techniques that can simultaneously capture both host and microbial genome-wide expression patterns while retaining the spatial information.

One recent article reported on the capture of both host and microbial genome-wide expression patterns simultaneously using germ-free plants and a synthetic microbial consortia.^28^ This approach represented a significant advance in the field, but there are opportunities to include spatial information, progress beyond bulk rRNA sequencing, and to move past needing genome-characterized isolates to trace expression patterns. Ideally, to investigate root–microbe interactions, plant and microbe expression patterns could be simultaneously captured and spatially localized. But, due to the complexities involved in simultaneous expression capture in combination with spatial localization information from individual cells, separate approaches are currently necessary when seeking to capture more than just the rRNA expression of microbes. In lieu of comprehensive profiling methods to investigate root–microbe interactions, we suggest separately examining: (i) the response of single bacterial cells to host exudation within a population and at the community level, and (ii) dissecting how individual root cells respond to their immediate surroundings and environmental perturbations.

Studying plant root expression, particularly in relation to root–microbe interactions, can result in the loss of environmental information, including microbial expression and host–microbe interactions. Despite the application of single-cell RNA sequencing (scRNA-seq) techniques across a diverse range of plant tissues and species, these methods have yet to effectively capture and maintain the root–microbe interface. A potential solution to overcome this limitation could be to focus on root exudates to quantify host–microbe interactions. By encapsulating exudates and individual bacterial cells within gel-bead droplets, it would then be possible to assess within population responses of bacterial cells to these exudates. Capturing exudates under various stress treatments could be pertinent to understand bacterial adaptation and changes in function and physiology in relation to host–environment interactions, which could have implications for C usage and storage.

Exploring microbial activity and composition at a high resolution can also be achieved using single-cell RNA-seq and spatial transcriptomics, but as mentioned above, currently needs to be captured independently from host expression. In the past few years, there has been an emergence in new bacterial single-cell RNA-seq approaches, with the ability to capture genome-wide expression from thousands of individual bacteria.^29^ In the context of the rhizosphere, using bacterial single-cell RNA-seq could identify expression heterogeneity from any bacteria of interest, including those directly involved with C and N nutrient cycling. Exploring the spatial expression patterns of microbes at the single-cell scale has also been achieved. Despite a technical limitation of approximately 100 marker-genes per single cell, par-seqFISH^30^ can capture expressed transcripts from different microbes, which can also be monitored over time. For example, selecting key C and N genes from different microbes of interest could potentially uncover previously unknown influences involved with nutrient cycling over time.

## Conclusion

Getting to the root of root–microbe interactions requires a synthesis of ecophysiology, microbiology, ecology, and emerging technologies. It is becoming increasingly clear that separating fine roots according to functionality is important when modelling C dynamics and examining root–microbe relationships. In our previous studies, we have shown that microbial dynamics are different according to fine root functionality, and there is now scope to apply this thinking to ecosystem functions and use cutting-edge sequencing techniques to decipher spatial patterns. Understanding the functional processes occurring at each fine root environment is vital to contribute to our understanding of ecosystem functions. We would like to end this commentary with a quote from McCormack et al. (2015): “Compared with their closest above-ground analogs, functional divergence between absorptive and transport fine roots would be equivalent to that observed between leaves and twigs” and if I were studying the leaf microbiome, the picture certainly wouldn’t be any clearer by mixing in a few twigs.

## References

[1] King WL Yates CF Cao L , et al. Functionally discrete fine roots differ in microbial assembly, microbial functional potential, and produced metabolites. Plant Cell Environ 2023; 46: 3919–3932. 37675977 10.1111/pce.14705

[2] Berendsen RL Pieterse CMJ Bakker PAHM . The rhizosphere microbiome and plant health. Trends Plant Sci 2012; 17: 478–486. 22564542 10.1016/j.tplants.2012.04.001

[3] Reinhold-Hurek B Bünger W Burbano CS , et al. Roots shaping their microbiome: global hotspots for microbial activity. Annu Rev Phytopathol 2015; 53: 403–424. 26243728 10.1146/annurev-phyto-082712-102342

[4] Ling N Wang T Kuzyakov Y . Rhizosphere bacteriome structure and functions. Nat Commun 2022; 13: 36. 35149704 10.1038/s41467-022-28448-9 PMC8837802

[5] Hiltner L . Über neuere Erfahrungen und Probleme auf dem Gebiet der Bodenbakteriologie und unter besonderer Berücksichtigung der Gründüngung und Brache. Arbeiten der Deutschen Landwirtschaftlichen Gesellschaft 1904; 98: 59.

[6] Kuzyakov Y Razavi BS . Rhizosphere size and shape: temporal dynamics and spatial stationarity. Soil Biol Biochem 2019; 135: 343–360.

[7] Edwards J Johnson C Santos-Medellín C , et al. Structure, variation, and assembly of the root-associated microbiomes of rice. Proc Natl Acad Sci U S A 2015; 112: E911. 10.1073/pnas.1414592112 PMC4345613 25605935

[8] McCormack ML Dickie IA Eissenstat DM , et al. Redefining fine roots improves understanding of below-ground contributions to terrestrial biosphere processes. New Phytol 2015; 207: 505–518. 25756288 10.1111/nph.13363

[9] Pregitzer KS DeForest JL Burton AJ , et al. Fine root architecture of nine North American trees. Ecol Monogr 2002; 72: 293–309.

[10] Cannon WA . A tentative classification of root systems. Ecology 1949; 30: 542–548.

[11] Wilcox HE . Morphological studies of the root of red pine, *Pinus resinosa* I. Growth characteristics and patterns of branching. Am J Bot 1968; 55: 247–254.

[12] Pregitzer KS . Fine roots of trees – a new perspective. New Phytol 2002; 154: 267–270. 33873419 10.1046/j.1469-8137.2002.00413_1.x

[13] Freschet GT Pagès L Iversen CM , et al. A starting guide to root ecology: strengthening ecological concepts and standardising root classification, sampling, processing and trait measurements. New Phytol 2021; 232: 973–1122. 34608637 10.1111/nph.17572 PMC8518129

[14] Ostonen I Truu M Helmisaari H-S , et al. Adaptive root foraging strategies along a boreal–temperate forest gradient. New Phytol 2017; 215: 977–991. 28586137 10.1111/nph.14643

[15] Guo D Mitchell RJ Withington JM , et al. Endogenous and exogenous controls of root life span, mortality and nitrogen flux in a longleaf pine forest: root branch order predominates. J Ecol 2008; 96: 737–745.

[16] Fleishman SM Eissenstat DM Bell TH , et al. Functionally-explicit sampling can answer key questions about the specificity of plant–microbe interactions. Environ Microbiome 2022; 17: 51. 36221138 10.1186/s40793-022-00445-x PMC9555203

[17] Herms CH Hennessy RC Bak F , et al. Back to our roots: exploring the role of root morphology as a mediator of beneficial plant–microbe interactions. Environ Microbiol 2022; 24: 3264–3272. 35106901 10.1111/1462-2920.15926 PMC9543362

[18] Schaffer-Morrison SAZ Zak DR . Mycorrhizal fungal and tree root functional traits: strategies for integration and future directions. Ecosphere 2023; 14: e4437.

[19] Kwatcho Kengdo S Peršoh D Schindlbacher A , et al. Long-term soil warming alters fine root dynamics and morphology, and their ectomycorrhizal fungal community in a temperate forest soil. Glb Chg Bio 2022; 28: 3441–3458. 10.1111/gcb.16155 35253326

[20] King WL Yates CF Guo J , et al. The hierarchy of root branching order determines bacterial composition, microbial carrying capacity and microbial filtering. Commun Biol 2021; 4: 83. 33875783 10.1038/s42003-021-01988-4 PMC8055976

[21] Winter G Todd CD Trovato M , et al. Physiological implications of arginine metabolism in plants. Review. Front Plant Sci 2015; 6: 534. 26284079 10.3389/fpls.2015.00534 PMC4520006

[22] Xiong Y Fan P Fu S , et al. Slow decomposition and limited nitrogen release by lower order roots in eight Chinese temperate and subtropical trees. Plant Soil 2013; 363: 19–31.

[23] Goebel M Hobbie SE Bulaj B , et al. Decomposition of the finest root branching orders: linking belowground dynamics to fine-root function and structure. Ecol Monogr 2011; 81: 89–102.

[24] Krishna MP Mohan M . Litter decomposition in forest ecosystems: a review. Energy Ecol Environ 2017; 2: 236–249.

[25] Yates C King WL Richards SC , et al. Temperate trees locally engineer decomposition and litter-bound microbiomes through differential litter deposits and species-specific soil conditioning. New Phytol 2024; 243: 909–921. 10.1111/nph.19900 38877705

[26] Saarenpää S Shalev O Ashkenazy H , et al. Spatial metatranscriptomics resolves host–bacteria–fungi interactomes. Nat Biotechnol 2023. doi:10.1038/s41587-023-01979-2 PMC11392817 37985875

[27] Lötstedt B Stražar M Xavier R , et al. Spatial host–microbiome sequencing reveals niches in the mouse gut. Nat Biotechnol 2023. doi:10.1038/s41587-023-01988-1 PMC11392810 37985876

[28] Vannier N Mesny F Getzke F , et al. Genome-resolved metatranscriptomics reveals conserved root colonization determinants in a synthetic microbiota. Nat Commun 2023; 14: 8274. 38092730 10.1038/s41467-023-43688-z PMC10719396

[29] Homberger C Barquist L Vogel J . Ushering in a new era of single-cell transcriptomics in bacteria. microLife 2022; 3: uqac020. 10.1093/femsml/uqac020 PMC10117829 37223351

[30] Dar D Dar N Cai L , et al. Spatial transcriptomics of planktonic and sessile bacterial populations at single-cell resolution. Science 2021; 373: eabi4882. 10.1126/science.abi4882 PMC8454218 34385369

